# Comparative Evaluation of Bioactive Synthetic NovaBone Putty and Calcified Algae-derived Porous Hydroxyapatite Bone Grafts for the Treatment of Intrabony Defects

**DOI:** 10.5005/jp-journals-10005-1379

**Published:** 2016-12-05

**Authors:** Nitika N Bembi, Sumit Bembi, Jyoti Mago, Gurpreet Kaur Baweja, Parvinder Singh Baweja

**Affiliations:** 1 Reader, Department of Periodontia, Shaheed Kartar Singh Sarabha Dental College and Hospital, Ludhiana, Punjab, India; 2 Professor, Department of Endodontia, Shaheed Kartar Singh Sarabha Dental College and Hospital, Ludhiana, Punjab, India; 3 Senior Lecturer, Department of Oral Medicine and Radiology, Shaheed Kartar Singh Sarabha Dental College and Hospital, Ludhiana, Punjab India; 4 Reader, Department of Prosthodontics, Guru Nanak Dev Dental College and Hopsital, Patiala, Punjab, India; 5 Reader, Department of Endodontia, Shaheed Kartar Singh Sarabha Dental College and Hospital, Ludhiana, Punjab, India

**Keywords:** Bone grafts, Bone regeneration, Intrabony defects.

## Abstract

**Introduction:**

To compare and evaluate clinically and radio-graphically the bone regeneration and the amount of bone fill in intrabony component of periodontal osseous defects through the osteoconductive and osteostimulative effect of bioactive synthetic NovaBone Putty - CMF and osteoconductive effect of calcified algae-derived porous hydroxyapatite Frios^®^ Algi-pore^®^ bone grafts.

**Materials and methods:**

Twenty-two sites in 11 patients, within the age range of 25 to 60 years, showing intrabony defects were selected according to split mouth design and divided into group I (Frios^®^ Algipore^®^) and group II (NovaBone Putty - CMF). All the selected sites were assessed with the clinical and radiographic parameters like plaque index, gingival index (full mouth and site specific), sulcus bleeding index, probing pocket depth, clinical attachment level, gingival recession, and radiographic bone fill. All the clinical and radiographic parameter values obtained at different intervals (baseline, 3, and 6 months) were subjected to statistical analysis.

**Results:**

A statistically significant reduction in pocket depth of 2.55 ± 0.52 mm (group I), 2.64 ± 0.67 mm (group II) and gain in clinical attachment level of 7.55 ± 1.44 mm (group I), 7.55 ± 2.38 mm (group II) were recorded at the end of the study. A slight increase in gingival recession was observed. The mean percentage change in amount of radiographic bone fill of group II (71.34%) was more than group I (61.93%).

**Conclusion:**

Both NovaBone Putty - CMF and Frios^®^ Algipore^®^ improve healing outcomes and lead to a reduction of probing depth, a resolution of osseous defects, and a gain in clinical attachment, but radiographic observation found better results with NovaBone Putty.

**How to cite this article:**

Bembi NN, Bembi S, Mago J, Baweja GK, Baweja PS. Comparative Evaluation of Bioactive Synthetic NovaBone Putty and Calcified Algae-derived Porous Hydroxyapatite Bone Grafts for the Treatment of Intrabony Defects. Int J Clin Pediatr Dent 2016;9(4):285-290.

## INTRODUCTION

Regeneration of the lost periodontium is one of the main goals of periodontal therapy. Regeneration of the periodontium must include the formation of new cemen-tum with inserting collagen fibers on the previously periodontitis-involved root surfaces and the regrowth of the alveolar bone.^1^ Conventional periodontal treatments, such as scaling and root planning are highly effective at repairing disease-related defects and halting the progression of periodontitis. However, they do little to promote regeneration of the lost periodontium. On the contrary, periodontal surgery, in particular regenerative periodontal surgery, aims not only to eliminate pocket depths but also to regenerate a new attachment apparatus and reconstruct the periodontal unit within previously existing normal physiologic limits.^2^

The effort to find a means to regenerate the peri-odontium has created a renaissance of research in the utilization of autogenous, allogenic, and alloplastic bone replacement materials in the treatment of periodontal osseous defects. A myriad of choices continue to increase as new materials are developed.

The ideal bone graft material should be able to trigger osteogenesis, cementogenesis, and a functionally oriented periodontal ligament at a more coronal level of attachment to the root surface. Most of the bone substitutes are osteoconductive, inert filling materials, and integrate with new bone. Osteoconductive materials provide a scaffold to allow in growth and deposition of bone.^3^

The use of nonautogenous bone replacement “grafts” for the treatment of intrabony defects has gained acceptance among clinicians, as it eliminates the need for intra-or extraoral bone graft donor sites.^4^ Several alloplastic materials are available today and these are synthetic substances used to fill bone defects. The goal is to fill the defect so that bone can adhere to the exterior surface of the implant material, infiltrate the interstices through pores, or biodegrade in advance of osteogenesis.^2^

In the quest to restore lost attachment, a variety of synthetic bone substitutes have been investigated. Recent innovations have suggested a substantial role of a bio-active glass on bone regeneration in periodontal osseous defects. Bioactive glass, a biocompatible product, has a positive influence on osteoblast culture and inhibitory capacity on fibroblast proliferation and on the apical migration of the junctional epithelium.^5^ The replacement of bioactive glass particles by new bone occurred due not only to an osteoconductive property, but also to an osteostimulatory capacity.^6^

The natural bone substitute Algipore^®^ has also been evaluated, which showed osseous formation, xenograft degradation, and bone ingrowth into particles.^7^ Thus, the present study was aimed to evaluate and compare clinically and radiographically the bone regeneration efficacy of NovaBone Putty - CMF and Frios^®^ Algipore^®^ bone grafts for the treatment of intrabony defects.

## MATERIALS AND METHODS

### Study Population and Design

Twenty-two sites in 11 patients (6 males and 5 females) between 25 and 60 years of age were selected according to the split mouth design study and divided into group I (Frios^®^ Algipore^®^) and group II (NovaBone Putty - CMF). The study was in accordance with the Helsinki Declaration of 1975, as revised in 1983, and all participants signed informed consent forms.

Each patient selected for the study satisfied the following criteria: (i) No medical problems that would contra-indicate routine periodontal surgery; (ii) patients with at least two intrabony defects, one in each quadrant or contra lateral side of the same arch with radiographic evidence of vertical/angular bone loss at affected sites; (iii) without any known allergy/hypersensitivity to any product used in the study; (iv) teeth not exhibiting grade III mobility.

The patients selected were subjected to assessment of plaque index (PI),^8^ gingival index (GI),^9^ and sulcus bleeding index (SBI).^10^ The probing depth, clinical attachment level,^11^ and gingival recession were recorded using the occlusal stent, UNC-15 periodontal probe/Gutta-percha points.^12^ These measurements were assessed at baseline, 3, and 6 months.

Intraoral periapical radiographs were taken for all selected 22 sites, using the long-cone paralleling technique to standardize the projection geometry, in order to measure the defect depth and defect fill both pre- and postoperatively. Standardized IntraoralPeriapica (IOPA) radiographs were scanned at 600 dpi using a digital scanner (HP Scanjet 3010 series scanner) and then imported to a laptop computer for further analysis. The images were then analyzed using the Image J 1.34S software program (National Institutes of Health). The radiographic assessment was carried out by analyzing the linear distances from the cemento-enamel junction (CEJ) to the base of the defect and from CEJ to the crest of alveolar bone.^13^ The difference between CEJ to the crest of alveolar bone and CEJ to the base of defect was considered as the amount of bone defect, and the difference between baseline to different intervals was considered as the amount of bone fill.^13^

Before the surgical treatment, patients received initial periodontal therapy with oral hygiene prophylaxis, professional tooth cleaning, and scaling.

### Surgical Protocol

After the presurgical evaluation and satisfactory response to phase I therapy, patients were subjected to surgical protocol under aseptic conditions. The operative site was anesthetized with 2% xylocaine HCl with adrenaline (1:80,000). The envelop flap was raised by giving intracre-vicular incisions extending at least one tooth mesial and distal to the intrabony defect, using Bard Parker knife with blade no. 12. The mucoperiosteal flap was raised using periosteal elevator. The lining pocket epithelium was removed so that a fresh connective tissue bed was in contact with the graft material, and utmost care was taken to preserve the interdental papilla. This was done in order to allow better coverage of the graft material interproximally, to prevent exposure and exfoliation of the graft, and to aid in better healing. A thorough debride-ment was carried out in all the defect areas by using Gracey and universal curettes. Roots were planned and conditioned using 24% ethylenediaminetetraacetic acid (EDTA; at neutral pH).^14^

In group I, osteoconductive bone graft Frios^®^ Algipore^®^ was mixed in dappen dish with patient’s blood to get a cohesive mass and was placed into the defect site. In group II, NovaBone Putty - CMF in the form of ready to use was placed directly into the defect site (Fig. 4). The graft placed in the defect was secured in place with the approximation of flaps using presuturing technique, and surgical area was protected with noneugenol dressing (Coe-Pack, GC America Inc, Alsip, IL, USA).

All patients were prescribed systemic doxycycline HCl 200 mg for the first day followed by 100 mg/day for 5 days, and a combination of ibuprofen (400 mg) and paracetamol (500 mg) thrice daily for 5 days.^15^ Patients were instructed to rinse with chlorhexidine digluconate (0.2%) mouthwash twice daily for 2 weeks, and the patients were discharged with postoperative instructions.

### Statistical Analysis

The clinical and radiological data were evaluated using a commercially available statistical and power analysis software program. (Statistical Package for the Social Sciences (SPSS) 11.0, Chicago, IL, USA).

The arithmetic mean and standard deviations were calculated for the requisite assessment intervals. For the intragroup comparisons Wilcoxon signed-rank test was used, and for intergroup comparisons Mann–Whitney U test was applied.

**Table 1:** **Table 1:** Mean and mean differences in probing pocket depth, clinical attachment level and gingival recession of groups I and II at different intervals

				*Probing pocket depth*								*Clinical attachment level*								*Gingival recession*							
		*Assessment* * interval*		*Mean± SD*		*Mean* * difference* * from baseline*		*z value*		*p-value*		*Mean ± SD*		*Mean* * difference* * from baseline*		*z value*		*p-value*		*Mean± SD*		*Mean* * difference* * from baseline*		z *value*		*p-value*	
Group I		Baseline		6.45 ±1.44		*-*		*-*		*-*		11.27 ± 1.27		*-*		*-*		*-*		5.73 ±1.19		*-*		*-*		*-*	
		3 month		3.82 ± 0.87		2.63 ± 0.67		2.99		0.003		8.81 ±1.25		2.45 ± 0.82		2.98		0.003		5.90 ±1.45		0.18 ±0.40		1.41		0.16	
		6 month		2.55 ± 0.52		3.90 ±1.22		2.99		0.003		7.55 ±1.44		3.73 ±1.35		2.99		0.003		5.91 ±1.45		0.18 ±0.40		1.41		0.16	
Group II		Baseline		7.27 ± 2.37		*-*		*-*		*-*		11.55 ±3.24		*-*		*-*		*-*		5.55 ± 0.93		*-*		*-*		*-*	
		3 month		4.18 ±1.25		3.09 ±1.22		2.97		0.003		9.09 ±2.66		2.45 ±1.03		2.96		0.003		6.18 ±1.33		0.64 ± 0.67		2.33		0.02	
		6 month		2.64 ± 0.67		4.64 ±1.80		2.97		0.003		7.55 ±2.38		4.00 ±1.48		2.95		0.003		6.27 ±1.42		0.73 ± 0.79		2.27		0.02	

## RESULTS

A total of 22 sites in 11 patients, within the age range of 25 to 60 years, showing intrabony defects were treated. Clinical evaluation of postsurgical healing revealed a good soft tissue response to the combinations with no adverse complications.

After 6 months, a significant reduction in probing dept (PD) from 6.45 ± 1.44 to 2.55 ± 0.52 mm (p = 0.003) was recorded in group I. In group II, significant reduction in PD from 7.27 ± 2.37 to 2.64 ± 0.67 mm (p = 0.003) was recorded.

**Fig. 1:**
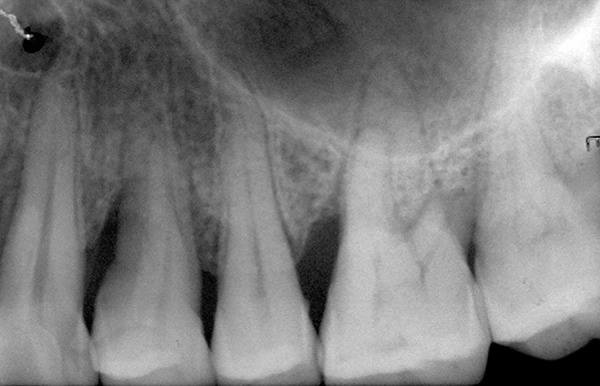
Baseline

**Fig. 2:**
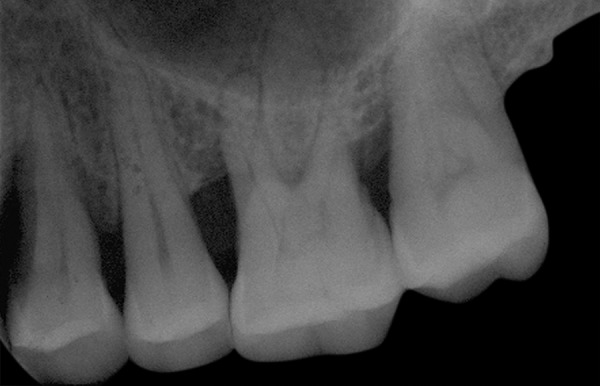
After 6 months using Algipore

The clinical attachment loss (CAL) was significantly reduced after 6 months from 11.27 ± 1.27 to 7.55 ± 1.44 mm (p = 0.003) in group I and from 11.55 ± 3.24 to 7.55 ± 2.38 mm (p = 0.003) in group II. After 6 months, the increase in gingival recession (GR) in group I was 0.18 ± 0.40 mm (p = 0.16) and in group II was 0.73 ± 0.79 mm (p = 0.02) (Figs 1 and 2; Table 1).

**Table 2:** **Table 2:** Mean and mean differences in probing pocket depth, clinical attachment level and gingival recession between groups I and II at different intervals

				*Probing pocket depth*								*Clinical attachment level*								*Gingival recession*							
*Assessment interval*		*Groups*		*Mean± SD*		*Mean* * difference* * (A-B)*		*z value*		*p-value*		*Mean ± SD*		*Mean* * difference* * (A-B)*		*z value*		*p-value*		*Mean ± SD*		*Mean* * difference* * (A-B)*		z *value*		*p-value*	
Baseline - 3 months		1		2.64 ± 0.67		0.45 ± 0.42		0.70		0.48		2.45 ±0.82		0.00 ±0.39		0.04		0.97		0.18 ±0.40		0.45 ± 0.24		1.79		0.07	
		II		3.09 ±1.22								2.45 ±1.04								0.64 ±0.67							
Baseline - 6 months				3.91 ±1.22		0.73 ± 0.66		0.85		0.39		3.73 ±1.35		0.27 ±0.60		0.38		0.71		0.18 ±0.40		0.55 ± 0.27		1.85		0.06	
		II		4.64 ±1.80								4.00 ±1.48								0.73 ±0.79							

**Table 3:** **Table 3:** Mean, mean differences, and percentage of amount of bone fill (radiographically) between groups I and II at different intervals

*Amount of bone fill (radiographically)*																	
*Assessment interval*		*Groups*		*Mean ±SD*		*Mean difference (A-B)*		*z value*		*p-value*		*Bone fill (%)*		z *value*		*p-value*	
Baseline - 3 months		I		1.16±0.70		0.13 ±0.26		0.83		0.41		39.42 ±7.12		1.81		0.07	
		II		1.29 ±0.52								45.27 ± 5.32					
Baseline - 6 months		I		1.79 ±0.98		0.23 ±0.37		1.02		0.31		61.94 ±7.66		2.96		0.006	
		II		2.02 ± 0.76								71.34 ±6.27					

**Fig 3:**
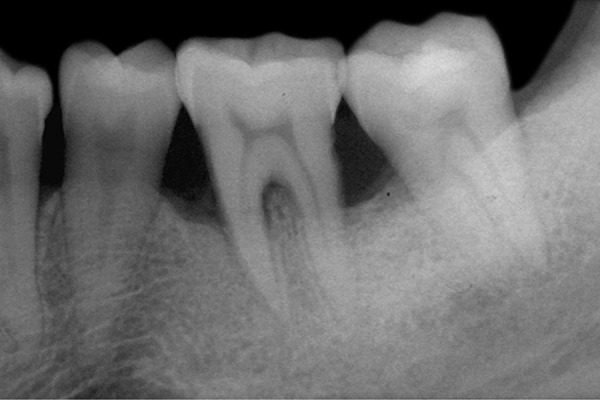
Baseline

**Fig 4:**
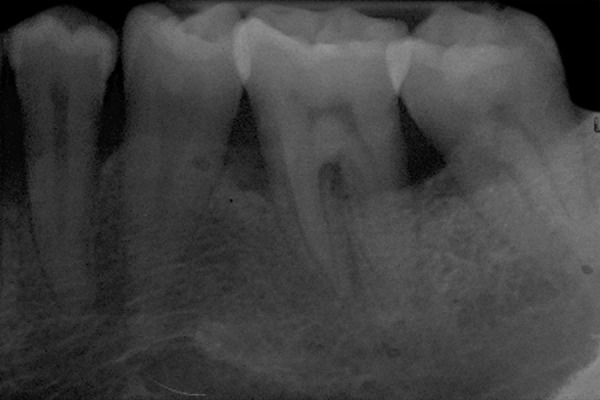
After 6 months using novapore

The statistical comparison of clinical parameters revealed no significant difference between the two groups (Figs 3 and 4; Table 2).

Analysis of the radiological parameters revealed a mean percentage change in the amount of radiographic bone fill of 61.94 ± 7.66% (p = 0.003) in group I and 71.34 ± 6.27% (p = 0.003) in group II. The intergroup differences were statistically significant, which indicate percentage change in radiographic bone fill was more in group II than group I (Table 3).

## DISCUSSION

Frios® Algipore® (Friadent, Mannheim, Germany) is a biological hydroxyapatite derived from porous-apatite of lime-encrusted ocean algae. Granules demonstrate a bone-equivalent microarchitecture and stoichiometry. Its chemical composition is pure inorganic calcium phosphate. The large specific surface area (50 m^2^/gm) and the high interconnecting microporosity of the particles should stimulate vascularization and bone ingrowth.^16^

NovaBone Putty – CMF is a bioactive synthetic graft with osteostimulative and osteoconductive property, manufactured by NovaBone, Florida, available in putty consistency. It consists of two particle phases: Phase 1 -90-710 μ bioactive glass particles and Phase 2 - 32-125 μ calcium phosphosilicate. Phase 2 particles enhance the physical characteristics and improve handling. Its putty consistency makes it easy to manipulate and adapts well to defects. Spaces between particles permit rapid vascularization and bone ingrowth. Bone forms in several areas in the defect simultaneously, thus enhancing the regeneration.

Kenney et al^17^ found a statistically significant reduction in probing pocket depth and gain in CAL on evaluation of a porous hydroxyapatite implant in periodontal defects. Stahl et al^18^ studied 12 infrabony periodontal lesions receiving surgical debridement followed by site implantation of porous hydroxyapatite implants. Clinical observations indicated a reduction in pocket depth consisting of both recession and clinical gain of attachment. Histological examination of the treated sites showed ossification of the implant pores and the implant periphery. They also found that this graft material offers the potential for increasing new bone mass within a human infrabony lesion Bowen JA et al.^19^

Turhani et al^20^ conducted a study to find the interaction between osteoblast-like cells isolated from man-dibular bone and hydroxyapatite ceramic bone substitute obtained from calcified red algae to assess the growth and differentiation of adherent cells on this biomaterial. The results of this study showed that hydroxyapatite ceramic bone substitute support the proliferation and differentiation of human osteoblast-like cells on its surface *in vitro* and might be suitable for use as scaffolds in tissue engineering strategies *in vivo.*

Studies on bioactive glass were also done. But, this is the first study to compare bioactive synthetic NovaBone Putty and calcified algae-derived porous hydroxyapatite bone grafts for the treatment of intrabony defects. In one study, bioactive glass was directly compared to a conventional flap procedure. A significantly higher attachment gain (1.5 mm) and a higher reduction in PD (0.8 mm) were observed after the use of the bioactive glass. A systematic review stated that mean difference in clinical attachment level gain between bioactive glass and open flap debridement alone was 1.05. It was also inferred that bioactive glass resulted in improvement of bony lesion when compared to open flap debridement.^21^ Another study was carried out to evaluate glass particulates in the periodontal osseous defects of 12 patients. There was a mean probing depth reduction of 3.33 mm, a mean attachment gain of 1.92 mm, and a mean radiographic bone fill of 3.47 mm. The authors also noted that ease of handling and excellent tissue responses were characteristic features of this material.^22^

The results of the present study show that treatment of intrabony defects with both group I (Frios^®^ Algipore^®^) and group II (NovaBone Putty - CMF) leads to significant PD reduction, CAL, and clinical and radiographic bone gain compared to baseline values. Percentage of bone fill with NovaBone Putty - CMF showed better results than Frios^®^ Algipore^®^.

We also found no antigenic or inadvertent reactions or tissue responses during the course of the study, indicating the safety of Frios^®^ Algipore^®^ and NovaBone Putty - CMF as clinical materials.

## CONCLUSION

The findings of this study reveal that both Frios^®^ Algipore^®^ and NovaBone Putty - CMF bone graft materials are biocompatible and safe to use without causing any inadvertent tissue response or antigenic reaction for the treatment of intrabony defects. There was no significant difference in the clinical outcome of the two materials, with a highly significant reduction in PD and gain in CAL. Radiographic observation revealed significant amount of bone fill and defect resolution with both the bone grafts, but based on the percentage of bone fill, NovaBone Putty - CMF showed better results. The degree of treatment success was dependent on good oral hygiene and inflammation-free periodontal tissue in the postoperative phase.

## ETHICAL CLEARANCE

By the ethical committee of the institute of Maharishi Markandeshwar College of Dental Sciences and Research (MMCDSR), where the study was conducted.
